# Analysis of Fire Resistance of Square-Cased Square Steel Tube Reinforced Concrete (ST-RC) Columns

**DOI:** 10.3390/ma14195541

**Published:** 2021-09-24

**Authors:** Gaoxiong Wang, Yanhong Bao, Li Yang, Yang Yu

**Affiliations:** 1 School of Civil Engineering, Qinghai University, Xining 810016, China; cewanggx@163.com (G.W.); ceyangl@163.com (L.Y.); 2 Qinghai Provincial Key Laboratory of Energy-Saving Building Materials and Engineering Safety, Qinghai University, Xining 810016, China; 3 Centre for Infrastructure Engineering (CIE), School of Engineering, Design and Built Environment, Western Sydney University, Penrith, NSW 2751, Australia

**Keywords:** square-cased square steel tube-reinforced concrete (ST-RC) columns, redistribution of internal forces, finite element model, duration of fire resistance

## Abstract

Based on the finite element (FE) analysis software Abaqus, an FE model of square-cased square steel tube reinforced concrete (ST-RC) columns under the hybridized action of high-temperature and load is established. The accuracy of the FE model is verified using experimental data from existing studies. This model is used to analyze the temperature change, internal force distribution, and failure characteristics of the square-cased square ST-RC columns under the action of fire, as well as the factors affecting the fire resistance limit of the column. The results of FE analysis show that under the action of fire, the maximum internal temperature of the square-cased square ST-RC columns occurs in the corner of the section. Moreover, the stress and strain reach their maximum values at the concrete corner outside the tube. During the heating process, an internal force redistribution occurs in the square-cased square ST-RC column. At the same time, the proportion of the axial force and the bending moment of the reinforced concrete outside the pipe decreases gradually, while the proportion of the internal force of the core concrete-filled steel tube (CFST) increases gradually. In essence, it is a process of load transfer from the high-temperature to the low-temperature zone. In addition, the section size, load ratio, slenderness ratio, cross-sectional core area ratio, steel content, and external concrete strength are the main parameters affecting the fire resistance limit of the square-cased square ST-RC columns. Among them, the cross-sectional core area ratio, section size, steel ratio, and external concrete strength are positively correlated with the fire resistance limit of the composite column. On the contrary, with the increase in the load ratio and the slenderness ratio, the fire resistance limit of the square-cased square ST-RC columns decreases. On this basis, a simplified formula to calculate the fire resistance limit of square-cased square ST-RC columns is proposed. The research results can be used as a theoretical reference for the fire protection design of this kind of structure in practical engineering.

## 1. Introduction

**Figure 1:**
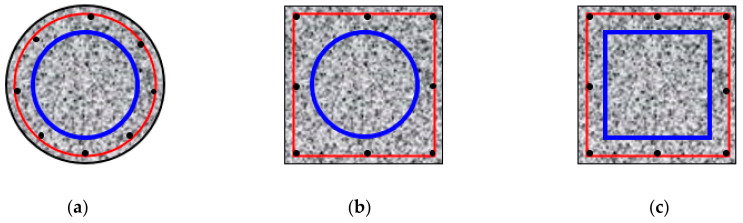
Section types of ST-RC columns (**a**) Circular section; (**b**) Square section;(**c**) Square-cased square section.

A steel-tube-reinforced concrete (ST-RC) column is a composite member formed by the combined action of core concrete-filled steel tube (CFST) components and peripheral reinforced concrete (RC) components. It combines the advantages of CFST columns and RC columns, and has excellent antiseismic, fire-resistant, explosion-resistant, and impact-resistant properties [1,2,3]. In practical engineering, composite columns are mainly used in high-rise and super-high-rise buildings. These ST-RC columns are generally categorized as round-cased circular columns, square-cased circular columns, and square-cased square ST-RC columns according to the geometric characteristics of the cross-section, as shown in Figure 1.

To date, many studies on the mechanical properties of ST-RC columns have been conducted. Yao et al. [4] calculated the axial compression and failure mechanism of ST-RC columns using a fiber model method, which contributed to the formula for calculating the axial compressive bearing capacity of ST-RC columns. Xu and Liu [5] studied the fire performance of four ST-RC columns and proposed a practical method for calculating the fire resistance performance of ST-RC columns. Xiang et al. [6] carried out fire experiments on square ST-RC columns and analyzed the effects of the slenderness ratio, load ratio, and section size on their refractor performance. Bao et al. [7] comprehensively analyzed the temperature change and internal force redistribution of ST-RC columns under fire using numerical simulation. Hou et al. [8] considered the effect of rising and falling temperature, and summarized the failure pattern and internal force distribution of ST-RC columns during the entire process of fire. Bao et al. [9] established an FE model of an ST-RC column-reinforced concrete beam plane frame under fire action, and explored the deformation law and internal force change of the plane frame under different beam load ratios. Xiang et al. [10] conducted experimental investigation on 20 composite short columns, and analyzed the bond-slip relationship between the steel pipe and concrete under and after high-temperature conditions. In addition, the effects of temperature, section shape, and other influencing parameters on the bond-slip properties were explored. Considering the effect of the maximum over fire temperature on the material degradation of SRC columns, Wang et al. [11] established an FE analysis model of SRC columns after fire. The model can be used to analyze the mechanical properties of SRC columns after fire. Wang et al. [12] analyzed the mechanical properties of an SRC frame in the stage of fire cooling, and revealed the changing law of section stress of specimens in the stage of fire cooling. However, the above studies mainly focused on the mechanical properties of ST-RC columns with round-cased and square-cased circular cross-sections. To date, few studies have been conducted on the fire resistance properties of ST-RC columns with square-cased square cross-sections, even though previous studies have shown that different cross-section forms have obvious effects on the temperature distribution of members, the internal force transfer, and the fire resistance limit [13,14,15,16,17].

In this study, to understand the working mechanism of square-cased square ST-RC columns under the combined action of fire and load, an FE model of this type of composite column was established through numerical simulation. The temperature change, stress, strain development, internal force distribution, and failure pattern of this kind of component in the process of fire heating were analyzed. Based on the parametric study of the factors affecting the fire resistance limit of this kind of column, a simplified calculation formula of the refractory limit of square-cased square ST-RC columns was developed.

## 2. FE Modeling

### 2.1. Introduction to FE Models

An FE model of square-cased square ST-RC columns under the combined action of fire and load was established by reasonably selecting the thermal parameters and mechanical model of the steel and concrete materials and adopting the thermo-mechanical sequential coupling method in the FE analysis software Abaqus. Using this model, the temperature distribution and mechanical properties of this kind of component were analyzed in detail.

### 2.2. Temperature Field Analysis Model

The temperature state of the column in a fire was simulated using the standard temperature rising curve of ISO-834 [18]. In the FE model of the temperature field, the eight-node, three-dimensional, solid heat transfer element DC3D8 was adopted for the steel pipe and the internal and external concrete members; the two-node heat transfer element DC1D2 was employed for the stirrups and the longitudinal reinforcement. Thermal radiation and convection occur between the concrete and air on the surface of the composite column in afire. The thermal convection coefficient is $25\;W/(m^{2}\cdot {}^{\circ}C)$ and the thermal radiation coefficient is 0.5 [19]. Without considering the thermal contact resistance between steel and concrete, it is assumed that they are completely heat-transferring. A tie restraint was adopted for the steel pipe and concrete, and reinforcement and concrete.

The thermal parameters proposed by Lie [20] were used to simulate the thermal properties of steel and concrete during the heating process under fire. Considering that water evaporation has a strong influence on the specific heat of concrete, Han [1] stated that the best simulation effect can be achieved when the moisture content is 5%, and the concrete specific heat formula is modified as follows:

(1) $$\left\{\begin{array}{l} \rho_{c}^{\prime }c_{c}^{\prime }=0.95\rho_{c}c_{c}+0.05\rho_{w}c_{w}\left(T\leq 100\;{}^{\circ}C\right)\\ \rho_{c}^{\prime }c_{c}^{\prime }=\rho_{c}c_{c}\left(T>100\;{}^{\circ}C\right) \end{array}\right.$$

where *ρ*_c_′ and *c*_c_′ and are the bulk density and specific heat of water vapor evaporation, respectively; *ρ*_c_ and *c*_c_ are the bulk density and specific heat without considering the evaporation of water vapor, respectively; *ρ*_w_ and *c*_w_ are the bulk density and specific heat of water, respectively.

### 2.3. Mechanical Analysis Model

Through the thermal-mechanical sequential coupling method, the temperature field calculation results were accurately introduced into the mechanical model to analyze the mechanical properties of the square-cased square ST-RC columns. In this model, the isotropic elastoplastic model is used in the steel constitutive model, which satisfies the von Mises yield criterion. The stress–strain relationship of steel under high temperature was obtained from Lie [20]. The plastic damage model is used in the constitutive model of concrete; the concrete inside the steel pipe adopts the constitutive model of core concrete at elevated temperature, which was put forward by Han [1] and considers the influence of ξ (the constraint effect). In addition, the stress–strain relationship of concrete outside the steel tube under high temperature was adopted from Lie and Denham [21]. The tensile strength of concrete adopts the relationship of tensile performance of concrete under high temperature, as proposed by Shi et al. [22], and the expression is given as follows:

(2) $$f_{t}^{T}=(1-\frac{T}{1000})f_{t}\left(20\;{}^{\circ}C\leq T<1000\;{}^{\circ}C\right)$$

**Figure 2:**
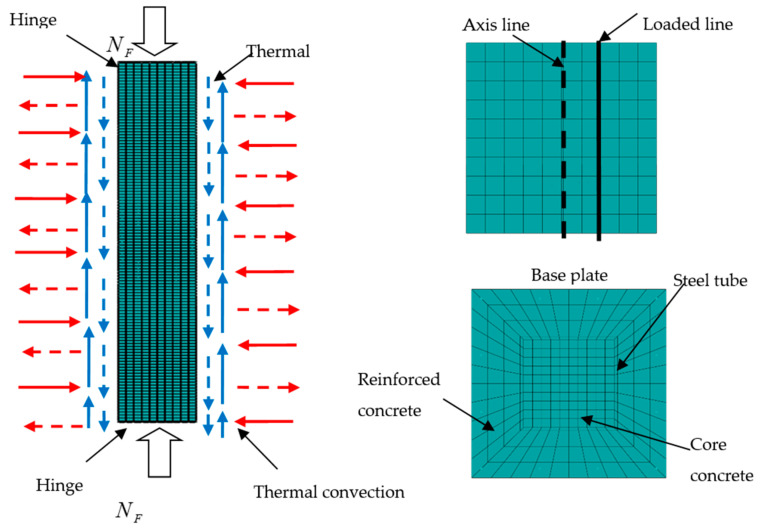
FE model of the square-cased square ST-RC columns.

The interface between the steel pipe and the internal and external parts of the concrete was modeled as a surface contact. Hard contact was used in the normal direction, in which the pressure can be transferred completely but the tension cannot. Coulomb friction was used tangentially, which can transfer shear force and allow the steel pipe and concrete to generate a relative slip, with a friction coefficient of 0.6 [1]. Stirrups and concrete, reinforcement and concrete were constrained by being embedded. The boundary conditions and the meshing of the square-cased square ST-RC columns are shown in Figure 2.

## 3. Validation of the FE Models

### 3.1. Verification of the Ultimate Bearing Capacity of the ST-RC Columns at Ambient Temperature

**Figure 3:**
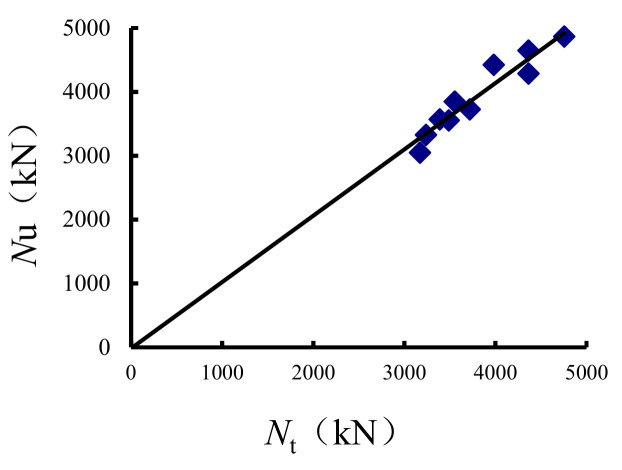
Ultimate bearing capacity simulation and comparison with the literature.

In this study, the axial compression and eccentric compression data of CC1-CC10 ST-RC columns from Kang at al. [23] were used for the simulation. Displacement loading was adopted, and the boundary was hinged at both ends. Figure 3 shows the comparison between the data from the literature and the simulation results, where, *N*_t_ and *N*_u_ are the experimental and calculated values of the ultimate bearing capacity, respectively. As shown in the figure, the correlation between the test and calculation results of the bearing capacity is 0.917, which shows that the test and calculation results agree well.

### 3.2. Verification of the ST-RC Columns under Fire

**Figure 4:**
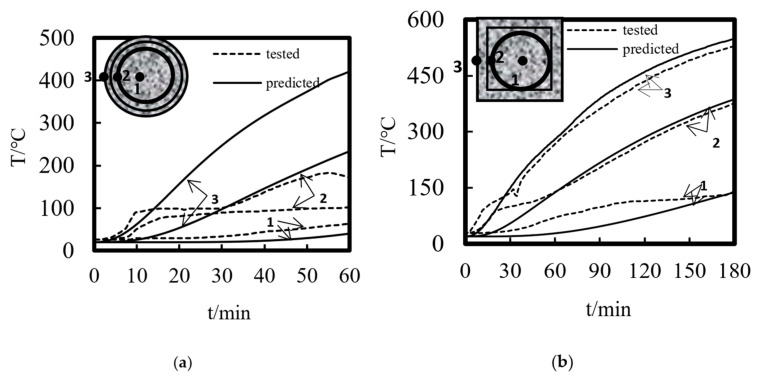

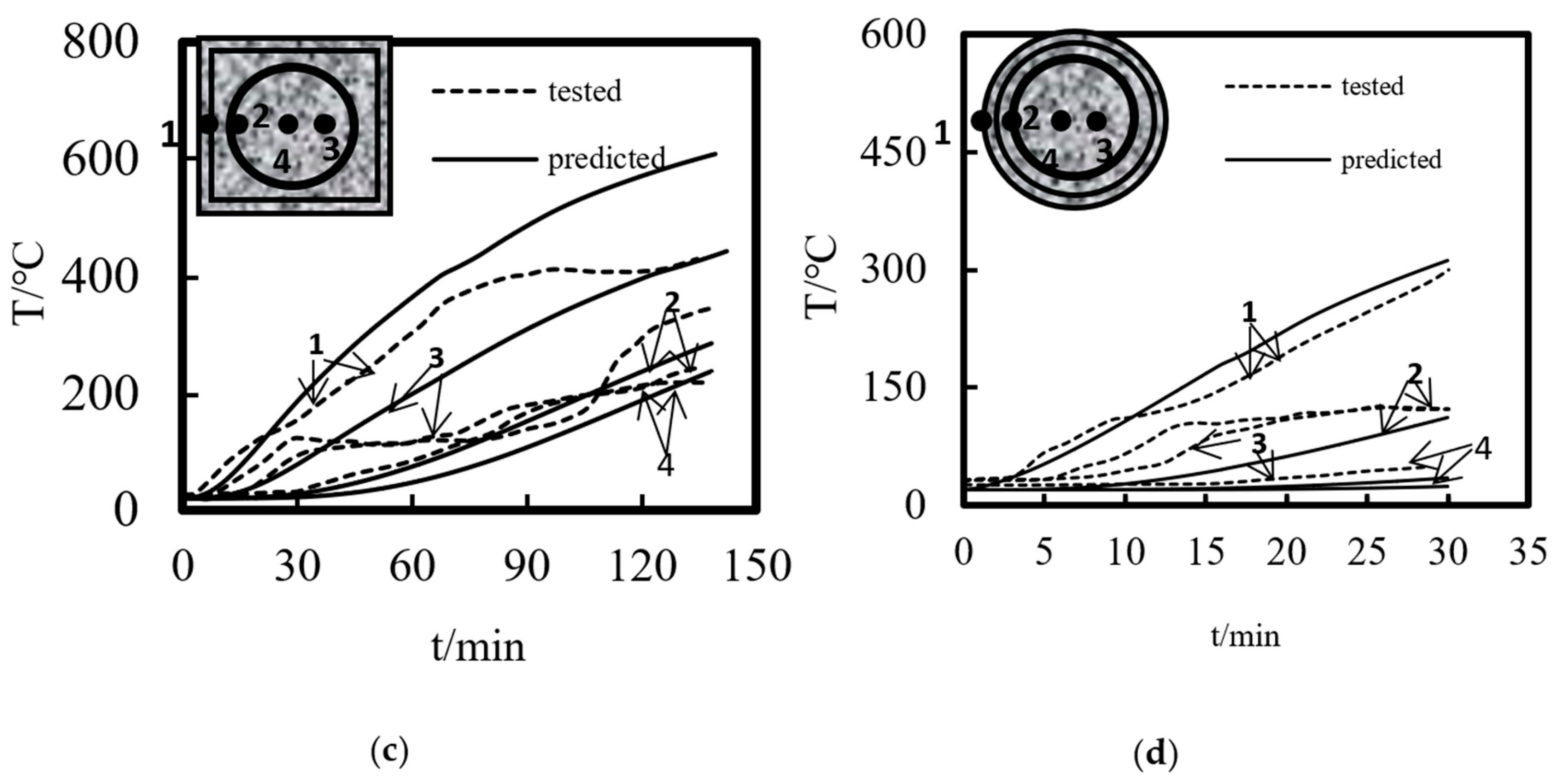
The comparison of temperature versus time curves of the ST-RC columns (**a**) C4; (**b**) S4; (**c**) SZ1-1; (**d**) CZ2-1.

**Figure 5:**
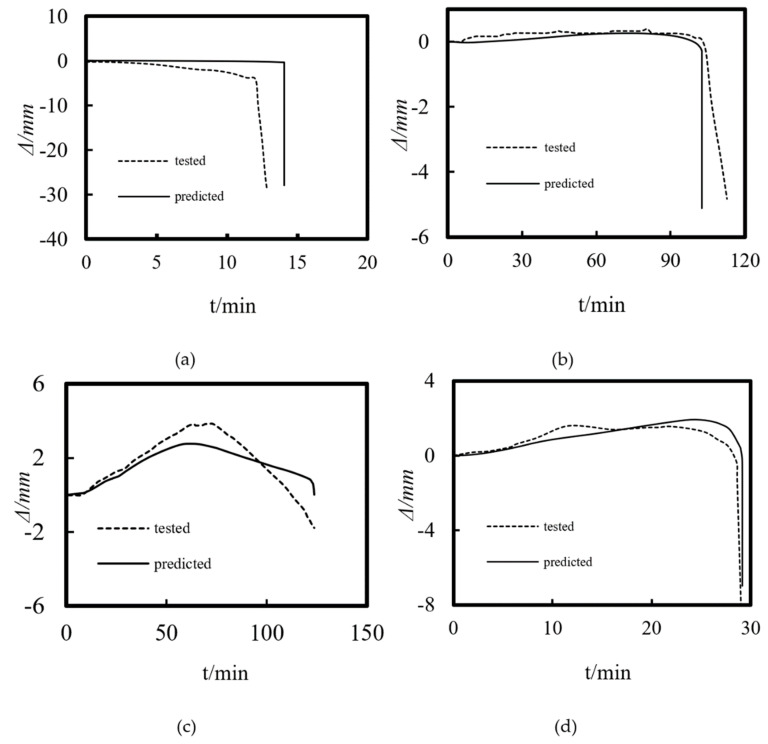
The comparison of the axial displacement versus time curves of the ST−RC columns (**a**) C4; (**b**) S4; (**c**) SZ1-1; (**d**) CZ2-1.

Using the above modeling method, the experimental results of Xu and Liu [5] and Xiang et al. [6] for the temperature distribution and axial displacement–time curve of ST-RC columns under fire were calculated and compared. The fire endurance test data and the calculated results are shown in Table 1. Figure 4 shows the comparison between the simulation results and the experimental data of the temperature field. As shown in the figure, the simulation curve of the temperature change of the cross-section is basically consistent with the experimental curve. The deviation of some measuring points may be caused by the movement of the temperature measuring points when pouring concrete in the process of making the specimen. Figure 5 shows a comparison between the experimental data and the calculated results of the axial displacement–time curves. It is clearly shown that the trend of the test curve is basically consistent with that of the calculated curve. The difference in the fire resistance limit is less than 10% in Table 1, indicating that the simulation results are in good agreement with the experimental values.

## 4. Analysis of Fire Resistance of the ST-RC Columns

With reference to the material grade and construction requirements specified in the *Technical Regulations for Concrete Stacking Collections* CECS188-2019 [24] and *Concrete Structural Design Specification* GB50010-2010 [25], a typical example of a square-cased square ST-RC column was established. The basic parameters are provided as follows: section size (*B*) is 600 mm, CFST outer diameter (*D*) is 300 mm, steel tube thickness (*t*) is 10 mm, and column height (*H*) is 6000 mm. The column is symmetrically equipped with 12Φ20 HRB400 longitudinal reinforcement, the HPB300 reinforcement with diameter of 8 mm is used as a stirrup, and the spacing is 100 mm. The steel pipe is made of Q390 steel and the yield strength is *f*_y_ = 390 Mpa. C40 self-compacting commercial concrete is used for both inner and outer concrete of the steel tube. Table 2 reports the mechanical properties of both the steel and concrete. According to the calculation, the slenderness ratio of the column *λ* = *L/B* = 10, the reinforcement ratio *ρ*_s_ is 0.14%, the core area ratio *α*_sc_ is 0.25, the steel content *α*_s_ is 0.15, and the column load ratio *n* is 0.6 (*N*_F_ is the axial force applied during fire and *N*_u_ is the ultimate bearing capacity of the member at room temperature). In addition, the initial eccentricity used to analyze the fire resistance limit of the square-cased square ST-RC columns was *L*/1000.

### 4.1. Temperature History

**Figure 6:**
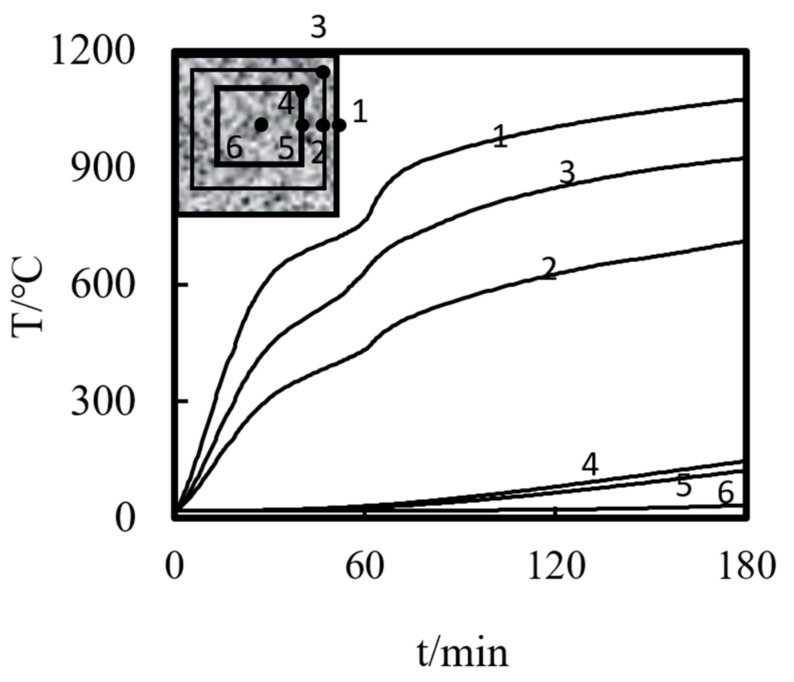
Temperature time–curve of each point in the cross-section.

Figure 6 shows the temperature–time variation curve of each characteristic point in the half-height section of the square-cased square ST-RC column. As shown in the figure, the temperature of each point in the section increases gradually with the increase in the heating time. By comparing the characteristic points, it can be seen that the temperatures of the corners of the concrete outside the pipe, the corners of the longitudinal bars, the corners of the steel tubes, and the corners of the core concrete are higher than those of the other positions of the same material at the same time. The temperature of the characteristic points on the section decreases gradually from the outside to the inside. Moreover, the temperature of the concrete inside the steel pipe is much lower than that of the concrete outside the pipe. This indicates that the heating rate at the corner of the square-cased square ST-RC column is the fastest under the action of fire. The existence of the external concrete effectively hinders the inward transfer of temperature and protects the internal concrete-filled steel tube.

**Figure 7:**
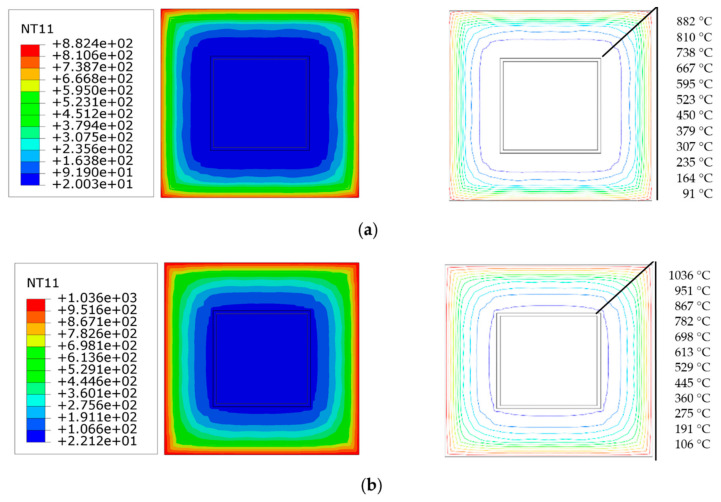

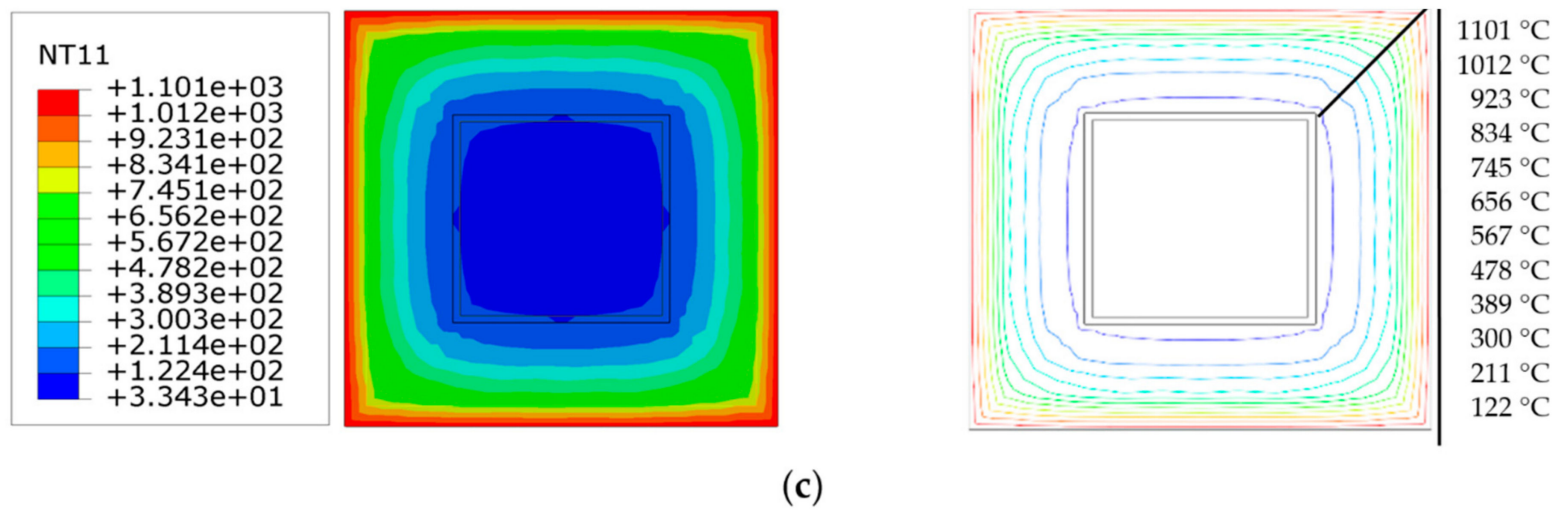
Temperature field distribution at different times (**a**) 60 min; (**b**) 120 min; (**c**) 180 min.

Figure 7 shows the time variation in the temperature field distribution in the half-height section of the square-cased square ST-RC column. As shown in the figure, the temperature decreases gradually from the fire surface to the center of the section. In addition, the isotherm changes from dense to sparse, indicating that the temperature difference changes faster at areas closer to the edge of the section. From 60 to 120 min, the isotherm at the edge of the section becomes thinner gradually, and the rate of change of the temperature difference decreases. From 120 to 180 min, the density of the isotherm at the edge of the section changes little, which indicates that the heating rate of the concrete outside the tube decreases gradually with the increase in the heating time. This corresponds to the temperature–time curve of the internal characteristic points of the section in Figure 6.

### 4.2. Failure Pattern

**Figure 8:**
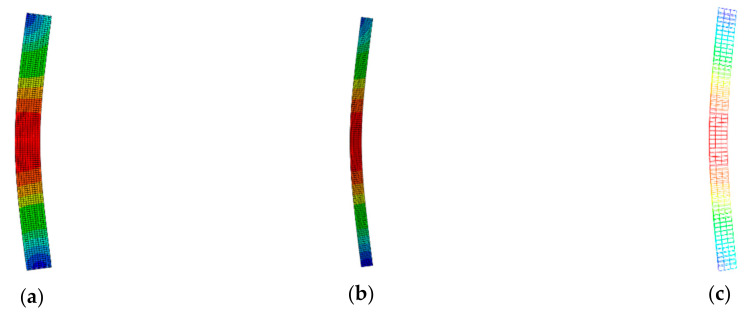
Failure mode (**a**) The overall damage form; (**b**) CFST; (**c**) Steel reinforcement cage.

Figure 8 shows the failure pattern of the square-cased square ST-RC columns when exposed to fire. As can be seen in the Figure 8, due to the existence of the core CFST, the ST-RC columns have good deformation capacity. Therefore, only the overall instability and failure of the member occur, and local buckling deformation is absent.

### 4.3. Axial Displacement–Time Curves

**Figure 9:**
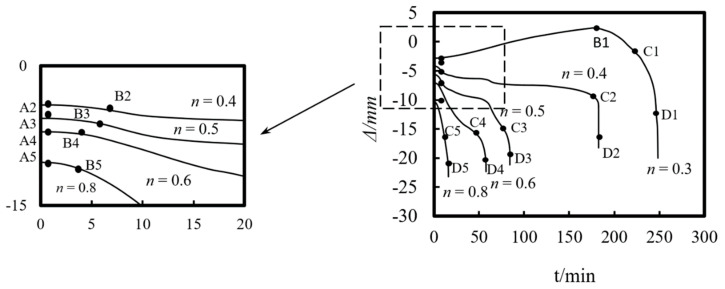
Axial deformation−time curves under different fire load ratios.

Figure 9 shows the axial displacement–time curves of the square-cased square ST-RC columns with different axial compression ratios. As seen in the figure, the displacement-time curve can be roughly divided into four characteristic stages:

(1) Normal temperature loading phase (O–A). In this stage, the axial compression of the upper load causes the initial axial compression deformation of the composite column. The larger the load ratio, the greater the axial compression displacement.
(2) Temperature expansion stage (A–B). During the initial temperature increase, with the decrease in the load ratio, the increasing rate of the axial displacement compression at the top of the column decreases gradually. When the load ratio is less than 0.3, the axial displacement is positive; that is, the axial expansion displacement is at the top of the column. The smaller the load ratio, the more obvious the axial displacement reduction and the longer the temperature rise expansion phase (A–B). This is due to the simultaneous expansion and degradation during the initial heating process. When the load is relatively large, the material expansion is the dominant factor affecting axial displacement because the heating time is short and the section temperature is lower than the degradation temperature of concrete (450 °C) and steel (200 °C). Therefore, the axial displacement increase rate reduces. With the increase in the heating time, the expansion reaches its peak at point B. Since the upper load is large, the components have an internal force distribution. However, the resistance of the structure is less than the upper load, which causes the axial displacement to increase gradually. As a result, the duration of heating and the expansion of A–B is short. When the axial pressure is small, stage A–B lasts a long time. The main reason contributing to this phenomenon is that as the heating time increases, the internal temperature of the cross-section gradually increases, and the material properties also begin to deteriorate. However, due to the low axial pressure, the deformation caused by the axial compression is still less than that caused by heating and expansion. Until point B, the material degradation is obvious, showing a rapid decline in the composite column’s carrying capacity. Meanwhile, the axial displacement of the composite column begins to increase.
(3) Material softening stage (B–C). As the heating time increases, the internal temperature of the member increases gradually, and the material degradation dominates the axial deformation. When the axial pressure is relatively large, stage B–C lasts longer due to the shorter heating time at point B and the lower degree of material degradation. With the increase in the heating time, the component material gradually degrades until point C, where the failure begins to accelerate. When the axial pressure is relatively low, the duration of phase B–C is shorter. This is because the temperature rise at point B has been occurring for a long time and the internal temperature of the cross-section is high, resulting in obvious material degradation. The resistance of the structure is close to the upper load. When the temperature rise continues to point C, the column cannot support the axial load and begins to enter the accelerated failure stage.
(4) Accelerated failure stage (C–D). After point C, due to the existence of initial eccentricity and the degradation of the material properties, the column undergoes bending deformation, and the additional bending moment caused by the second-order effect increases rapidly, which leads to the sharp failure after point C.

### 4.4. Internal Force Distribution

**Figure 10:**
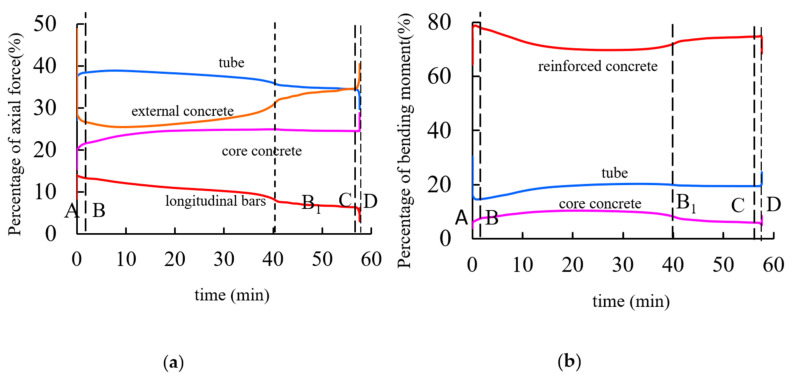
Percentage of internal force (**a**) Axial force ratio curve; (**b**) Moment ratio curve.

Figure 10a shows the variation curve of the percentage of the axial force to the total axial force of the external concrete, steel bar skeleton, steel pipe, and interior concrete of the square-cased square ST-RC column during the heating process when the load ratio is 0.6. As shown in the figure, when the O–A stage is loaded at ambient temperature, the axial forces of the external concrete, reinforcement cage, steel pipe, and interior concrete account for 38%, 15%, 27%, and 20% of the total axial force, respectively. In stage A–B, the expansion deformation plays a leading role, and the external reinforced concrete is loaded with a large load. In stage B–B_1_, the concrete material outside the pipe degrades with the increase in temperature. Meanwhile, the unloaded load is borne by the steel pipe and the longitudinal bar. However, with the increase in the heating time, the internal temperature of the steel pipe and the longitudinal bar increases gradually, and the material degradation leads to a decrease in the load. At this time, the unloaded load is transferred to the interior, which results in a gradual increase in the pressure on the core concrete. Point B_1_ is the critical point for the flexural deformation of the composite columns. Before point B_1_, the flexural deformation of the member is small, and it has little effect on the redistribution of the internal force of the member. However, after point B_1_, the member produces a large lateral displacement, which has an obvious influence on the redistribution of the internal force in the composite column. At the end of point B_1_, the axial forces of the external concrete, steel reinforcement cage, steel pipe, and interior concrete account for 30%, 8%, 38%, and 24% of the total axial force, respectively. In stage B_1_–C, due to the large flexural deformation, the second-order effect produces a large additional moment, but the robust compressive capacity of the concrete leads to an increase in the axial compression percentage of the internal and external concrete. When point C enters the accelerated failure stage, the axial forces of the external concrete, reinforcement cage, steel pipe, and interior concrete account for 35%, 5%, 35%, and 25% of the total axial force, respectively. Finally, the ST-RC column enters the accelerated failure stage C–D and the axial pressure on the internal and external concrete begins to rise rapidly. Meanwhile, the axial force on the steel decreases rapidly.

Figure 10b shows the variation curve of the percentage of the external reinforced concrete, steel pipe, and interior concrete to the total bending moment of the square-cased square ST-RC column during the heating process when the load ratio is 0.6. Due to the presence of the initial eccentricity, the member generates a small additional bending moment during the O–A normal temperature loading phase. At this moment, the bending moment of the reinforced concrete, steel pipe, and interior concrete account for 77%, 16%, and 7% of the total bending moment, respectively. In stages of A–B and B–B_1_, the properties of the external reinforced concrete materials gradually deteriorate with the increase in temperature. In addition, the axial pressure borne by the external reinforced concrete begins to decrease, and the percentage of the bending moment also reduces. Meanwhile, the steel pipe and the interior concrete experience increased axial force, and the percentage of the bending moment continuously increases. When the heating time reaches the critical point of flexural deformation at B_1_, the bending moments of the reinforced concrete, steel pipe, and interior concrete account for 70%, 21%, and 9% of the total bending moment, respectively. With continued heating, after point B_1_, due to the increasing transverse deflection and the bending of the column, the axial force of the peripheral concrete begins to increase and the additional moment caused by the second-order effect also becomes larger. As a result, the proportion of the affected bending moments also begins to increase. At the end of the B_1_–C stage, the bending moments of the reinforced concrete, steel pipe, and interior concrete account for 74%, 18%, and 8% of the total bending moment, respectively. In the accelerated failure stage C–D, the buckling deformation of the composite column increases sharply, and the lateral deformation of the surrounding concrete is serious. However, due to the existence of the internal concrete-filled steel tube, the column does not show local buckling and it has good bending resistance. Therefore, the increased bending moment is mainly transferred to the internal CFST. As a result, the proportion of the bending moment borne by the external reinforced concrete decreases, while that of the internal core concrete-filled steel tube increases rapidly.

According to the above analysis, from the end of loading at ambient temperature (O–A) to the stage of accelerated failure (C–D), the proportion of the axial force of the external reinforced concrete and the bending moment of the composite columns gradually decreases, while the proportion of the axial force of internal concrete-filled steel tube and the bending moment gradually increases. The results show that in the process of heating, the internal force of the square-cased square ST-RC column is redistributed, which is essentially the process of load transfer from the external high-temperature zone to the internal low-temperature zone.

### 4.5. Strain Development

**Figure 11:**
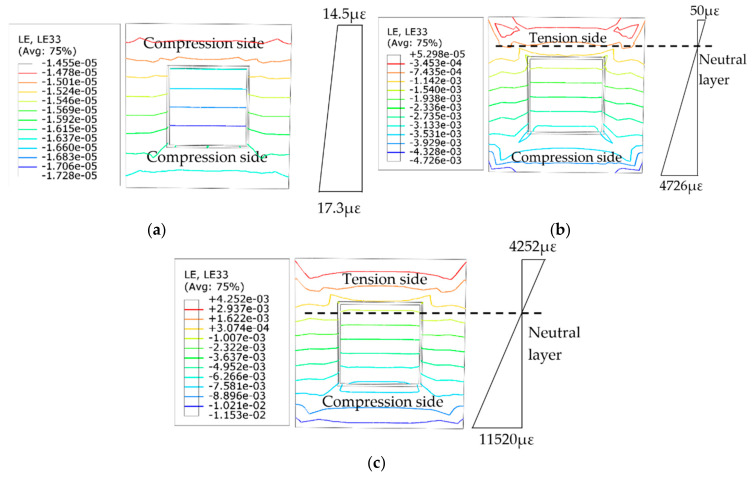
Strain distribution at different times of fire at the half-height section (**a**) *t* = 0 min; (**b**) *t* = 30 min; (**c**) *t* = 56 min.

Figure 11 shows the cross-sectional strain distribution results at the half-height of the square-cased square ST-RC columns at different moments of fire. The specified strain is positive in terms of cross-section tension and negative in terms of cross-section compression. In the initial stage, because there is only initial eccentricity, the cross-section has not been exposed to fire, and the material has not yet begun to degenerate; therefore, the entire cross-section is subjected to compression. Accordingly, the distribution of the cross-section strain is approximately isoline. With the increase in heating time, due to the degradation of the material’s properties, the transverse deflection increases, and the member begins to bend, resulting in a tensile section beginning to appear on the side far from the initial eccentricity (a section with a strain of zero). As shown in Figure 11b, when *t* = 30 min, the strain on the tension side is smaller, close to 1/90 of the compression side. At this moment, the strain isolines of the external concrete corner and the square concrete-filled steel pipe corner of the section are dense. The maximum strain also occurs in the corner area. When the temperature continues to rise, the internal material of the section begins to degrade with a significant increase in the component bending. As a result, the neutral layer moves toward the compression zone and the area of the tension zone increases rapidly. When the fire resistance limit reaches 58 min, the strain on the tension side is about one-half that on the compression side. At this time, the height of the tensile area is close to the height of the compression area, and the component is destroyed, as shown in Figure 11c.

The strain of the square-cased square ST-RC column increases continuously from the time of ambient temperature loading to the accelerated failure stage. However, the tensile strain increases faster than the compressive strain due to the weak tensile property of concrete. Moreover, in the outer concrete material, the cross-section angle strain is the maximum, which is caused by the higher temperature at the corner of the concrete outside the pipe and the rapid degradation of the material.

### 4.6. Stress Development

**Figure 12:**
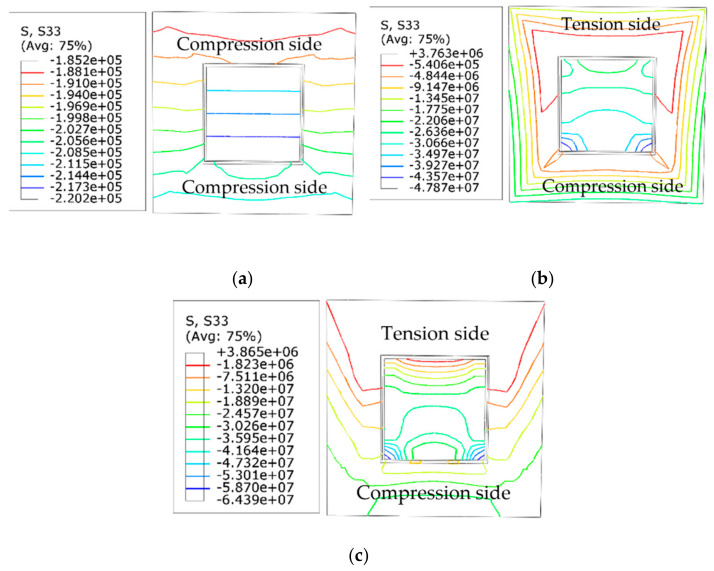
Stress distribution at different times of fire at the half-height section (**a**) *t* = 0 min; (**b**) *t* = 30 min; (**c**) *t* = 56 min.

Figure 12 shows the results of the stress development in the half-height section of the square-cased square ST-RC columns at different times of fire. The specified stress is positive in terms of cross-section tension and negative in terms of cross-section compression. In the loading stage at ambient temperature, due to the small initial eccentricity, the entire internal section is under compressive stress, and the distribution of the cross-section stress is approximately isoline. As the duration of exposure to fire increases, since the material degradation and the side direction deflection become large, tensile stress begins to appear in the cross-section. As shown in Figure 12b, when *t* = 30 min, the maximum tensile stress is only 1/10 of the compressive stress, and the isometric stress lines at the inner and outer concrete corners and steel tubes are dense. Meanwhile, the maximum stress occurs at the corners. When the temperature continues to rise, the height of the tension region begins to increase. At *t* = 58 min, the maximum tensile stress is only 1/20 of the compressive stress. At this time, the stress isoline is concentrated at the corner of the steel pipe and the interior and external concrete sections. Meanwhile, the height of the tension side is close to the compression side, and the member is destroyed rapidly, as shown in Figure 12c.

From the loading at ambient temperature to the accelerated failure stage, the internal stress of the square-cased square ST-RC columns increases continuously with the increase in the heating time. However, the growth rate of the compressive stress is faster than that of the tensile stress because the compressive performance of concrete is better than its tensile performance.

**Figure 13:**
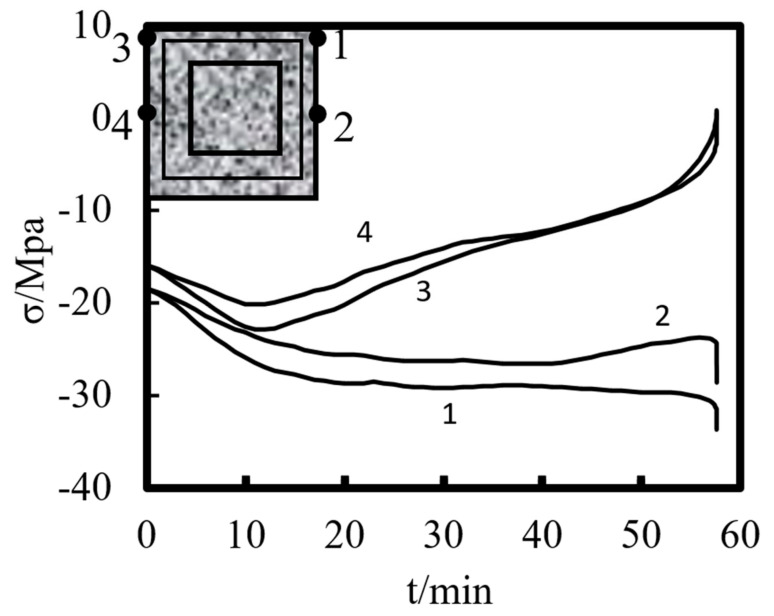
Stress–time curve of the external concrete at various points in the half-height section.

Figure 13 shows the stress–time curve of characteristic points on the external concrete of the half-height section of the square-cased square ST-RC column. Points 1 and 2 are the cross-sectional compression area of the concrete corners and the edge center characteristic points, respectively. As can be seen from the figure, with the increase in the heating time, the compressive stress of the concrete in the compression area increases continuously, but the increase in the rate becomes increasingly slower. This is because, during the temperature increase process, the cross-section is transversely displaced, resulting in a tensile force. As a result, the neutral axial compression zone moves, the tensile stress increases rapidly, and the compressive stress increases slowly. Points 3 and 4 are the characteristic points of the concrete’s corner and the edge center in the tension area of the mid-span section, respectively. As can be seen from the figure, the stress at the point in the tension zone is a compressive stress at the beginning of heating. As the rising time of temperature increases, the compressive stress first increases, then decreases, and finally becomes a tensile stress. This is because the initial eccentricity is smaller, and the entire section is pressed at the end of the normal temperature load phase. During the heating process, the side of the section far from the eccentric line changes from a compression zone to a tension zone. In the initial stage, the height of the compression zone gradually decreases, which leads to a continuous increase in the compressive stress. After forming the tension region, the compressive stress begins to decrease gradually due to the generation of the tensile stress. Moreover, with the increase in the height of the tension area, the tensile stress begins to increase. Comparing the characteristic points of the corner of the concrete section and the characteristic points of the central edge of the concrete section shows that the compressive stress and the tensile stress at the corner of the concrete section are greater than the compressive stress and tensile stress at the central edge of the section.

**Figure 14:**
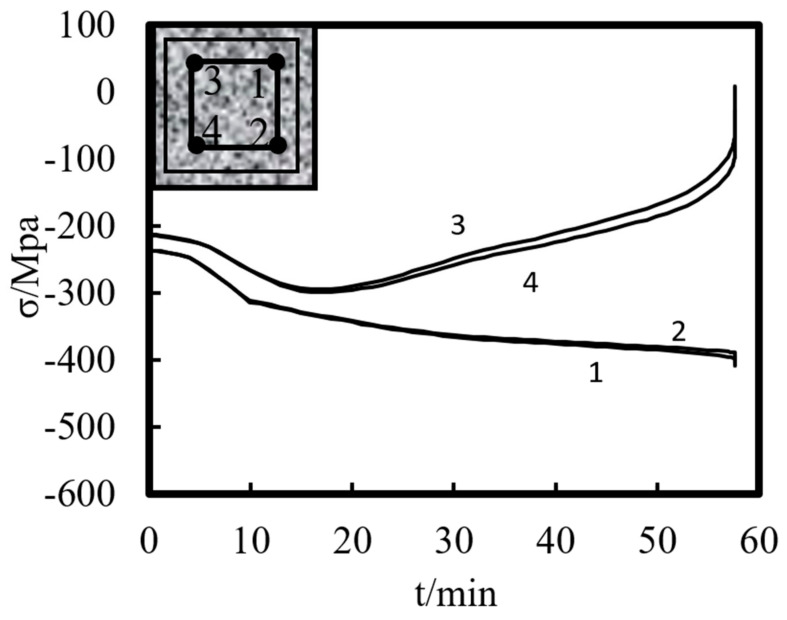
Stress–time curve of the internal steel tube at various points in the half-height section.

Figure 14 shows the stress–time curve of characteristic points on the steel tube of the half-height section of the square-cased square ST-RC column. Points 1 and 2 are the steel tube corners and edge center feature points, respectively, of the cross-sectional compression area. Similar to the stress change in the external concrete, with the increase in the heating time, the compressive stress of each characteristic point of the steel tube section increases continuously, but the increase in the rate decreases gradually. This is due to the transverse deflection of the composite column, which is generated on the tension side. Then, the tensile stress increases rapidly, while the compressive stress increases slowly. Points 3 and 4 are the characteristic points of the steel tube’s corner and edge center in the tension area of the mid-span section, respectively. These points show the same trend as the tensile stress of the concrete outside the pipe. The compressive stress first increases, then decreases, and subsequently becomes tensile stress. However, a comparison of the characteristic points shows that there is little difference between the stress of the corner point and the edge center point of the steel tube. This is because the external concrete impedes the transfer of temperature, resulting in a small difference between the temperature of the corner section and the edge center of the steel pipe, which effectively protects the internal steel pipe.

## 5. Parametric Analysis

**Figure 15:**
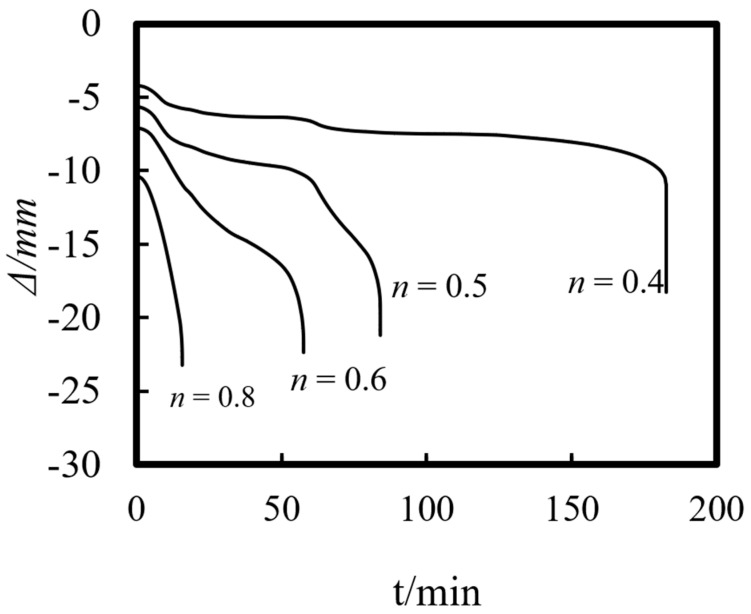
Effect of load ratio on the axial deformation–time curve.

**Figure 16:**
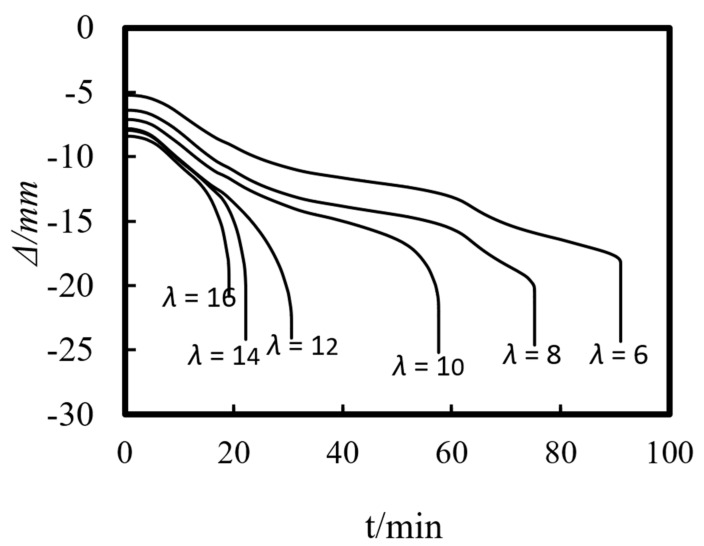
Effect of slenderness ratio on the axial deformation–time curve.

**Figure 17:**
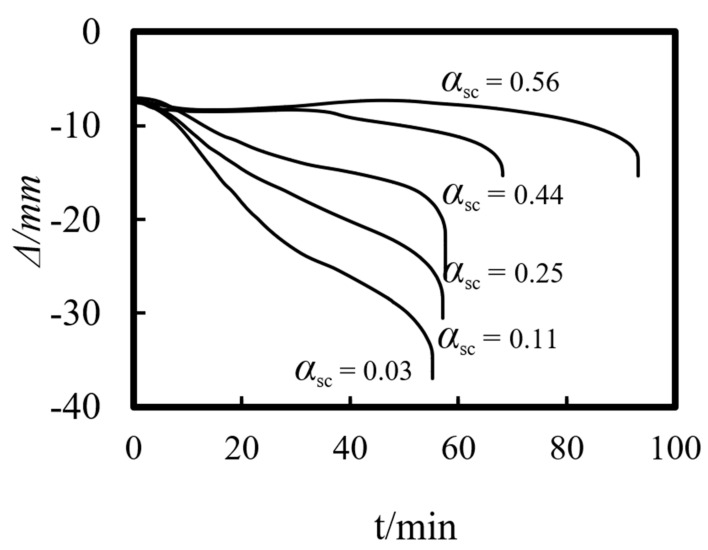
Effect of core area ratio on the axial deformation–time curve.

**Figure 18:**
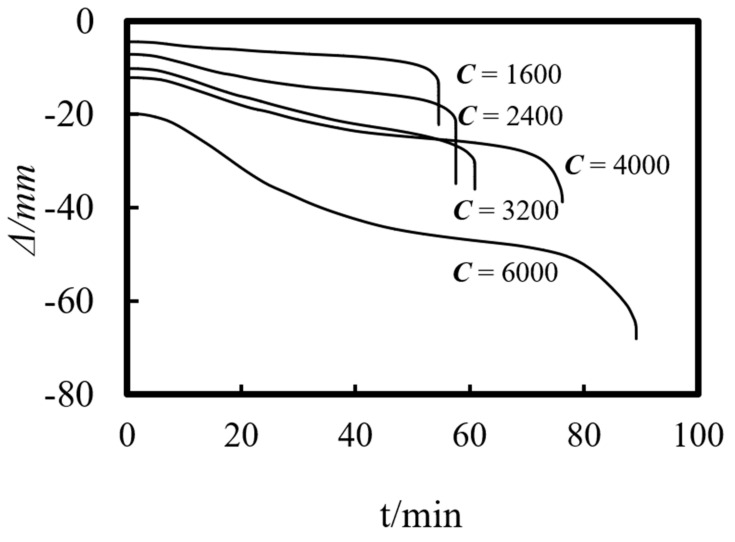
Effect of perimeter on the axial deformation−time curve.

**Figure 19:**
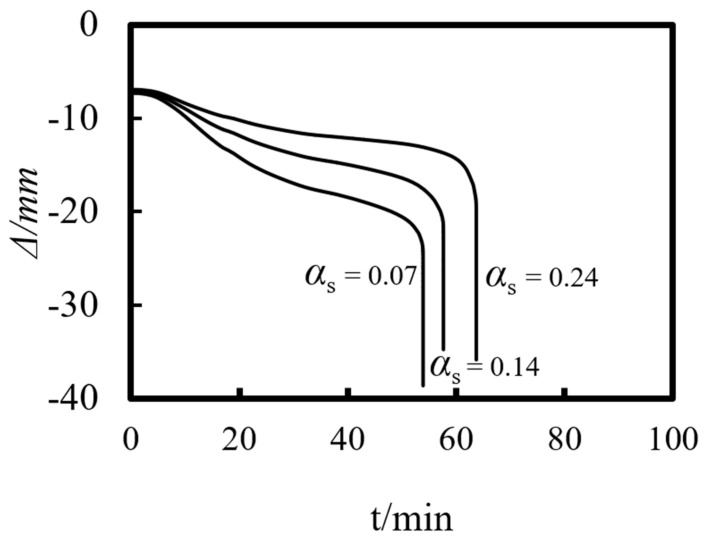
Effect of steel content on the axial deformation−time curve.

**Figure 20:**
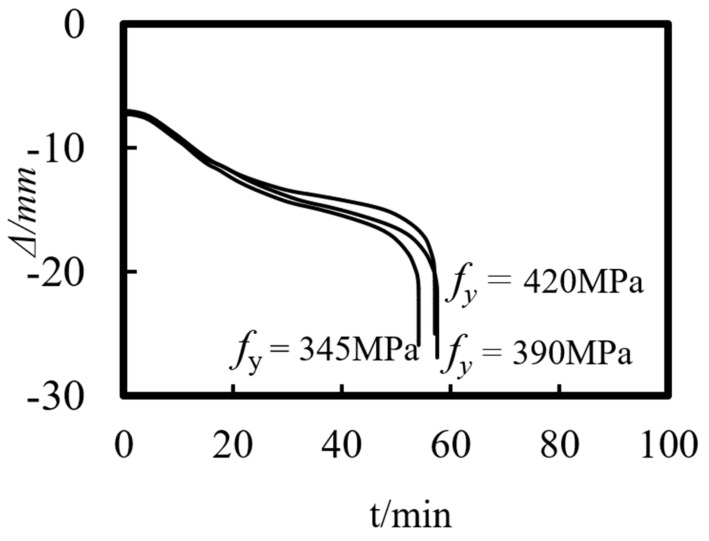
Effect of yield strength of steel tube on the axial deformation−time curve.

**Figure 21:**
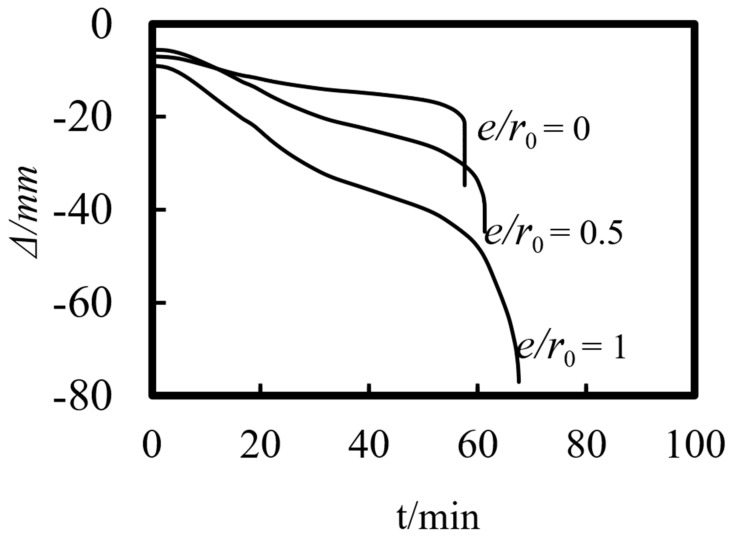
Effect of load eccentricity on the axial deformation−time curve.

**Figure 22:**
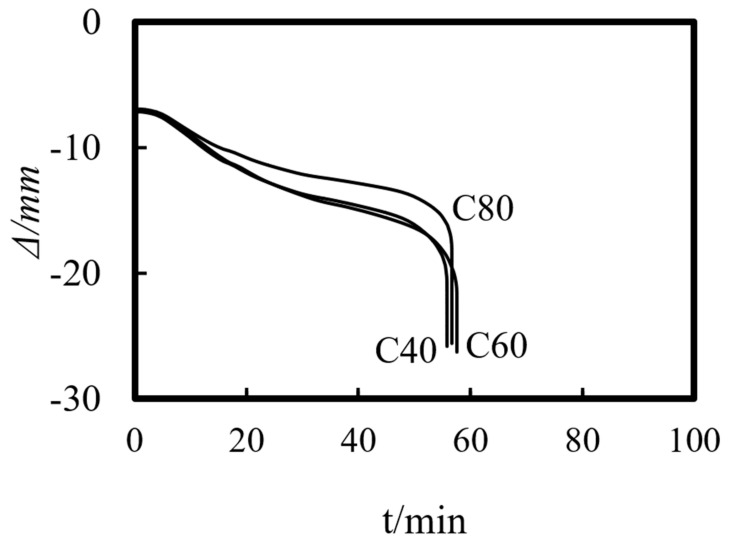
Effect of core concrete strength on the axial deformation−time curve.

**Figure 23:**
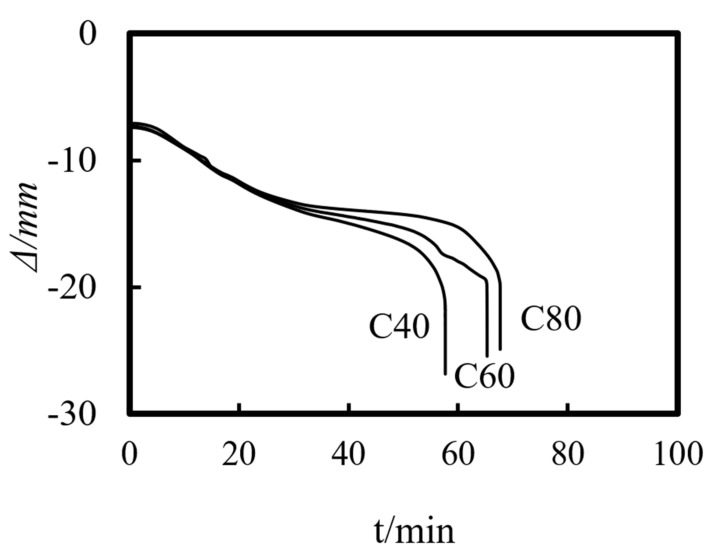
Effect of the external concrete strength on the axial deformation−time curve.

Previous studies on concrete-filled steel tube (CFST) members [26,27,28,29,30,31,32] showed that the main parameters affecting the fire resistance limit of ST-RC columns are section perimeter (*C*), fire load ratio (*n*), slenderness ratio (*λ*), cross-sectional core area ratio *α*_sc_ (*α*_sc_ = *A*_sc_/*A*_0_, where *A*_sc_ is the core concrete area and *A*_0_ is the area of the CFST), eccentricity *e/r*_0_(*r*_0_ = *B*/2), steel ratio (*α*_s_), yield strength of the steel tube (*f*_y_), yield strength of the steel bar (*f*_yb_), strength grade of the concrete (*f*_cu_), etc. The parameters of a typical example are given as follows: *C* = 2400 mm, *n* = 0.6, *λ* = 10, *α*_sc_ = 0.25, *α*_s_ = 0.15, *f*_y_ = 390 MPa, *f*_yb_ = 400 MPa, the strength grade of the concrete inside and outside the pipe is *f*_cu_ = 40 MPa, and the initial eccentricity is *L*/1000. Figure 15, Figure 16, Figure 17, Figure 18, Figure 19, Figure 20, Figure 21, Figure 22 and Figure 23 show the axial displacement–time curves of the square-cased square ST-RC column under the action of fire per theISO-834 standard, when the load ratio, slenderness ratio, cross-sectional core area ratio, section perimeter, steel content ratio, and concrete strength change.

### 5.1. Load Ratio (n)

Figure 15 shows the axial deformation–time curve of the composite column under different load ratios. As can be seen from the figure, the load ratio has a significant influence on the axial deformation of the ST-RC column. As the load ratio increases, the axial displacement of the member increases at the end of the loading stage at ambient temperature. Meanwhile, the axial deformation rate accelerates under the action of fire, and when the fire resistance limit is finally reached, the axial deformation of the member increases as well.

### 5.2. Slenderness Ratio (λ)

Figure 16 shows the axial deformation–time curve of the composite column with different slenderness ratios. The initial axial displacement of the ST-RC column increases with the increase in the slenderness ratio, and the axial deformation rate increases during the heating process. When the fire resistance limit is reached, the axial deformation of the composite column increases slightly when it is subjected to compression.

### 5.3. Cross-Sectional Core Area Ratio (α_sc_)

Figure 17 shows the effect of the cross-sectional core area ratio on the axial deformation–time curve of the composite column. It can be seen that the core area is less affected by the initial axial displacement, but it has a significant influence on the axial deformation during the failure of the member. This is because the CFST provides the main axial stiffness. The smaller the core area ratio, the smaller the total axial rigidity of the composite column. The material is degraded under the action of fire, and the axial stiffness of the member decreases obviously, which leads to the acceleration inthe axial deformation rate of the composite column during the process of heating. When the refractory limit damage is reached, the axial displacement of the composite column increases obviously.

### 5.4. Cross-Section of the Perimeter (C)

Figure 18 shows the effect of the section perimeter on the axial deformation-time curve of the composite column. As shown in the figure, when the section perimeter increases, the slenderness ratio of the column remains constant, but the height of the composite column increases in response. As a result, the axial displacement increases at the end of the loading stage at ambient temperature. Moreover, the axial displacement also shows an increasing trend during failure. This is because with the increase in the section size, the outer concrete area increases, resulting in a slow transfer rate of temperature to the interior of the column and a decrease in the rate of material degradation. Finally, when the composite column is destroyed, the upper load is applied for a long time, and the member undergoes large axial deformation.

### 5.5. Steel Content (α_s_)

Figure 19 shows the effect of steel content on the axial deformation-time curve of the composite column. As shown in the figure, the axial displacement at the initial stage is not large, but with rising temperature, the axial deformation rate of the ST-RC column increases with the decrease in the steel ratio. When the fire resistance limit is reached, the axial deformation of the member also increases gradually.

### 5.6. Yield Strength of the Steel Tube (f_y_)

Figure 20 shows the effect of the yield strength of the steel tube on the axial deformation–time curve of the composite column. As shown in the figure, the initial axial deformation of the member is independent of the yield strength of the steel tubes. As the yield strength of the steel pipe increases, the axial deformation rate of the column decreases during the heating process. However, the axial deformation remains unchanged when the fire resistance limit is reached.

### 5.7. Load Eccentricity (e/r_0_)

Figure 21 shows the axial deformation–time curve of the composite column under different eccentric loads. As can be seen in the figure, when the load eccentricity increases, the axial deformation during the initial loading phase increases correspondingly, resulting in an acceleration in the axial deformation rate during the heating process. When the refractory limit damage is reached, the axial displacement of the member increases significantly. This is because a larger initial eccentricity leads to a larger bending moment during the loading at room temperature. In the process of heating, the material’s properties deteriorate gradually, and the bending moment increases continuously under the influence of the second-order effect. As a result, the axial deformation rate of the member increases under the action of fire, and the axial displacement increases rapidly during the failure of the composite column.

### 5.8. Core Concrete Strength (f_cu_._in_)

Figure 22 shows the influence of the core concrete strength on the axial deformation–time of the composite columns. As shown in the figure, the core concrete strength has little effect on the axial deformation of the member under the combined action of load and temperature.

### 5.9. Strength of the Concrete Outside the Tube (f_cu_._out_)

Figure 23 shows the effect of the strength of the concrete outside the tube on the axial deformation-time curve of the composite column. As shown in the figure, when the strength grade of the concrete outside the tube decreases from C80 to C40, the axial deformation of the composite column increases slightly, when it reaches the fire resistance limit.

## 6. Practical Square-Cased Square ST-RC Column Fire Resistance Limit Calculation

**Figure 24:**
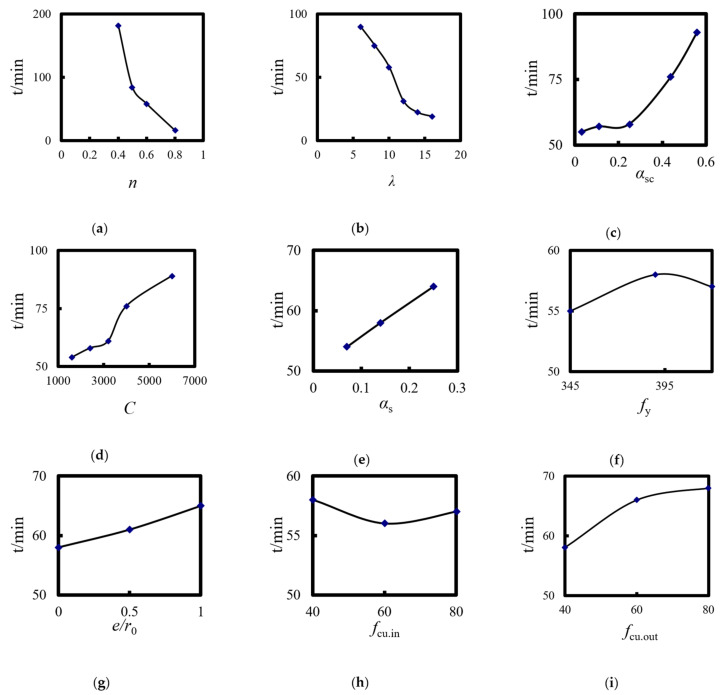
Effect of the parameters of the square-cased square ST-RC columns on the fire resistance limit (**a**) Load ratio; (**b**) Slenderness ratio; (**c**) Core area ratio; (**d**) Section perimeter; (**e**) Steel content; (**f**) Yield strength of steel pipe; (**g**) Load eccentricity; (**h**) Core concrete strength; (**i**) External concrete strength.

Figure 24 shows the effects of the square-cased square ST-RC columns’ main parameters on the fire resistance limit.

### 6.1. Load Ratio (n)

The load ratio is the main factor affecting the fire resistance of the composite column. When the load ratio is less than 0.4, the fire resistance limit of the member meets the requirement of the Eurocode for 3 h. As the load ratio increases, the fire resistance limit of the composite column begins to decrease significantly. When the load ratio is 0.8, the fire resistance limit of the member is only 15 min. Therefore, the upper load limit of composite columns should be strictly controlled in practical engineering applications.

### 6.2. Slenderness Ratio (λ)

The slenderness ratio has a significant influence on the fire resistance limit of the member. As the slenderness ratio increases, the ultimate bearing capacity and the fire resistance performance of the ST-RC column decrease correspondingly. As shown in Figure 24b, when the slenderness ratio increases from 6 to 16, the fire resistance limit reduces by 90 to 19 min. In addition, when the slenderness ratio is greater than 12, the reduction in the rate of the refractory limits of the composite columns gradually slows.

### 6.3. Cross-Sectional Core Area Ratio (α_sc_)

The cross-sectional core area ratio has a certain impact on the fire resistance limit of a composite column. As shown in Figure 24c, when the cross-sectional core area ratio is less than 0.25, it has little effect on the fire resistance limit of the member. However, as the core area increases, the overall stiffness of the column increases in response. As a result, the bearing capacity of the member as well as the fire resistance performance improve during the heating process.

### 6.4. Cross-Section of the Perimeter (C)

The section perimeter has an obvious effect on the fire resistance limit of the composite column. As the size of the member increases, the temperature absorption of the external concrete increases, which leads to a decrease in the temperature of the interior CFST. Finally, the fire resistance limit of the composite column increases significantly.

### 6.5. Steel Content (α_s_)

The impact of the steel content on the fire resistance limit of the composite column shows a linear increase. The higher the steel content and the thicker the steel pipe, the longer the member resists the fire.

### 6.6. Yield Strength of the Steel Tube (f_y_)

As the steel tube yield strength increases, the ultimate bearing capacity of the composite columns increases at ambient temperature, but it has little effect on the fire resistance performance of the members.

### 6.7. Load Eccentricity (e/r_0_)

As the load eccentricity increases and keeping the load ratio constant, the upper load decreases correspondingly, but it has little effect on the fire resistance limit of the composite columns.

### 6.8. Core Concrete Strength (f_cu_._in_)

The internal temperature fails to reach the core concrete’s material degradation temperature (450 °C) during the heating process, resulting in a lesser effect on the fire resistance limit of the composite columns.

### 6.9. Strength of the Concrete outside the Tube (f_cu_._out_)

With the increase in the heating time, the properties of the concrete material outside the pipe degrade. As the strength grade of the concrete outside the tube changes from C40 to C80, the fire resistance limit of the member changes from 58 to 68 min and the fire resistance limit increases.

The arrangement and analysis of the parameters of the square-cased square ST-RC columns show that the load ratio, slenderness ratio, core area, section perimeter, steel ratio, and concrete strength outside the tube are the main parameters affecting the fire resistance limit of the composite columns. Based on the analysis of the above parameters, a practical formula for calculating the fire resistance limit of square-cased square ST-RC columns under the action of an ISO-834 standard heating curve was designed, shown in Equation (3).

(3) $$\begin{array}{l} t_{R}=\left(1.23\lambda_{0}^{2}-5.89\lambda_{0}+6.90\right)\left(1.30\alpha_{\mathrm{sc}0}^{2}-1.62\alpha_{\mathrm{sc}0}+4.80\right)\left(0.390C_{0}^{2}+2.32C_{0}+5.76\right)\left(1.23\lambda_{0}^{2}-5.89\lambda_{0}+6.90\right)\\ \left(0.828\alpha_{s0}+4.34\right)\left(-1.21f_{\mathrm{cu}0}^{2}+4.48f_{\mathrm{cu}0}+0.311\right)/1.19e^{3.54n_{0}} \end{array}$$

where *t*_R_ is the fire resistance limit of the square-cased square ST-RC column, *n*_0_ = *n*/0.6, *λ*_0_ = *λ*/10, *α*_sc0_ = *α*_sc_/0.25, *C*_0_ = *C*/2400, *α*_s0_ = *α*_s_/0.14, and *f*_cu0_ = *f*_cu_/40. The application range of this formula combined with engineering examples is *n* = 0.3~0.8, *λ* = 6~16, *α*_sc_ = 0.05~0.5, *α*_s_ = 0.04~0.20, *C* = 1600~6000, *f*_cu_._in_ = 40~80 MPa, *f*_cu_._out_ = 40~80 MPa, and *f*_y_ = 235~420 MPa.

**Figure 25:**
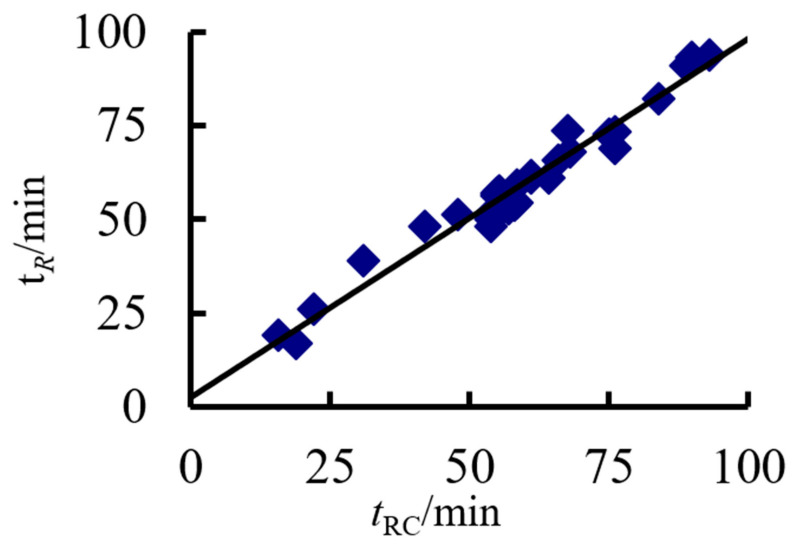
Comparison between the FE results and the simplified calculation results.

Figure 25 depicts a comparison of the practical calculations and numerical simulation results of square-cased square ST-RC columns. The *t*_R_ is the FE result and *t*_RC_ is the simplified calculation result of Equation (3). The correlation coefficient is 0.948, and, therefore, both results are in good agreement.

## 7. Conclusions

In this study, an FE model of square-cased square ST-RC columns under the combined action of fire and load was established. In addition, the changes in the temperature field, internal force distribution, stress, and strain during the failure of the composite columns were analyzed based on the results of previous studies. The main parameters affecting the fire resistance performance of the composite columns were investigated. Finally, a practical method to calculate the fire-resistance limit of the square-cased square ST-RC column was presented. The following are the conclusions in detail:

(1) Under the action of an ISO-834 standard heating curve, the internal temperature of the square-cased square ST-RC columns increases gradually. In addition, the temperature reaches its maximum in the corner area of the external concrete.
(2) The axial displacement–time curve of the square-cased square ST-RC columns under the action of fire can be divided into four characteristic stages. The load ratio has an obvious influence on the curve distribution of each stage.
(3) The variation curves of the internal axial force and the bending moment of the composite columns under the action of fire show that an internal force redistribution occurs during the fire process. In essence, it is a process of transferring heat from high-temperature zones to low-temperature zones.
(4) The load ratio, slenderness ratio, cross-sectional core area ratio, section perimeter, steel content, and concrete strength are the main parameters affecting the fire resistance limit of square-cased square ST-RC columns.
(5) A practical formula for calculating the fire resistance limit of square-cased square ST-RC columns was proposed, which was verified from the results of FE analysis. The good agreements between formula predictions and FE results indicate that the proposed formula can be utilized as a reference for practical engineering design.

## Figures and Tables

**Table 1:** Specimen parameters.

Specimen	*L* (mm)	*B* (mm)	*D* (mm)	*D*_s_ (mm)	*t*_s_ (mm)	*N*_0_ (kN)	*n*	Fire Resistance (min)	
								*t*	*t*_R_
SZ1-1	3810	300	-	203	6	1564	-	125	123
CZ1-1	3810	-	300	203	6	1553	-	29	28
C4	3650	-	330	219	4	-	0.85	13	14.8
S4	3650	330	-	219	4	-	0.5	112	102

**Table 2:** Mechanical properties of the materials.

Element	Material	*E*_m_ (MPa)	*v*	Compressive Strength (MPa)		Tensile Strength (MPa)		Ultimate Strength (MPa)
				*f*_ck_	*f*_y_	*f*_tk_	*f*_u_	*f*_st_
Tube	Q390	2 × 10^5^	0.3	-	390	-	490	-
Longitudinal reinforcement	HRB400	2 × 10^5^	0.3	-	400	-	400	540
Stirrup	HPB300	2 × 10^5^	0.3	-	300	-	300	420
Exterior concrete	C40	3 × 10^4^	0.2	26.8	-	2.39	-	-
Interior concrete	C40	3 × 10^4^	0.2	26.8	-	2.39	-	-

## Data Availability

The data presented in this study are available on request from the corresponding author.

## Nomenclature

*A*_sc_	core concrete area
*A*_0_	the area of the concrete-filled steel tube
*B*	cross-sectional width of ST-RC columns
*C*	section perimeter
*D*	diameter of circular ST-RC columns
*D*_s_	diameter of circular steel tube
*H*	cross-sectional height of rectangular ST-RC column
*N*_F_	axial compression load during the fire stage
*N*_u_	axial load-carrying capacity of columns at ambient temperature
*L*	length of ST-RC columns
*e/r*_0_	load eccentricity
*f*_cu_._in_	core concrete strength
*f*_cu_._out_	strength of the concrete outside the tube
*f*_y_	steel tube yield strength
*f*_yb_	reinforcement bars yield strength
*n*	load ratio of column
*t*_s_	thickness of steel tube
*t*	fire resistance of column
*t*_R_	the finite element result
*t*_RC_	the simplified calculation result of formula
*α*_s_	steel content
*α*_sc_	cross-sectional core area ratio
*λ*	slenderness ratio of column
*ρ*_s_	reinforcement ratio of column section
